# Data on plant defense enzyme activity associated with three endophytes against *Cornus florida Erysiphe pulchra* powdery mildew

**DOI:** 10.1016/j.dib.2023.109220

**Published:** 2023-05-11

**Authors:** Emily Rotich, Margaret T. Mmbaga

**Affiliations:** a South University in Nashville, 616 Marriott Dr. Nashville, TN 37214, USA; b Tennessee State University, 3500 John A Merritt Blvd. Nashville, TN 37209, USA

**Keywords:** Biological control, Disease management, Plant growth promotion, Reducing pesticides

## Abstract

Three bacteria endophytes that colonize flowering dogwood (*Cornus florida*) suppressed *Erysiphe pulchra* powdery mildew disease severity. The three bacteria identified as *Stenotrophomonas* sp. (B17A), *Serratia marcescens* (B17B), and *Bacillus thuringiensis* (IMC8) were assessed for plant defense enzymes associated with plant protection. Detached leaves inoculated with powdery mildew were spray treated with the selected bacterial isolates and incubated for 15 h, 26 h, 48 h and 72 h and then analyzed for activation of defense enzymes and Pathogenesis related (PR) proteins associated with induced systemic resistance (ISR) as a potential mode of action against powdery mildew. At each time point post treatment with the bacteria, leaf tissue was ground in liquid nitrogen and stored at -70°C for biochemical assay of enzyme activity. This data set presents the activation of enzyme activity for peroxidase (PO), polyphenol oxidase (PPO) and β -1,3-glucanase at 15 h, 26 h, 48 h and 72 h post treatment with bacteria as indicated by a change in absorbance min -1 mg-1 per gram fresh weight of leaves. The gene expression of the corresponding pathogenesis related (PR) protein for each bacterial treatment compared to the control was also analyzed using Real time PCR and five primers targeting PR1, PR2, and PR5. While changes for PO, PPO, and β -1,3-glucanase enzyme activities were observed at different time points post treatment with all three bacteria, expression of PR protein was detected for PR1, but it was negligible for PR2, and PR5.

**Specifications Table**

**Table:** 

Subject	*Agricultural Science*
Specific subject area	Evaluation of potential mechanism of action of three endophytes in *Cornus florida* plant protection against *Erysiphe pulchra* powdery mildew.
Type of data	Table Graphs: Analyzed data
How the data were acquired	Bacterial endophytes B17A, B17B, and IMC8 previously selected for suppressive activity against diverse fungal pathogens and as potential biological control agents for powdery mildew were evaluated for mechanism of action. A detached leaf assay was conducted in which leaves were spray treated with bacterial suspensions and incubated for 15 h, 26 h, 48 h and 72 h, and then analyzed for Peroxidase (PO), polyphenol oxidase (PPO), and β−1,3- glucanase enzymes as described by Rotich and Mmbaga (2022); Karthikeyan et al. (2005); and Pan et al. (1991) [[1], [2], [3]]. The gene expression of the corresponding pathogenesis related (PR) protein for each bacterial treatment was also analyzed using Real time PCR and five primers [4] and changes in amount of PR protein was calculated.
Data format	Tables and graphs ‘analyzed data’
Description of data collection	Data collection from 1 g leaf samples was homogenized in respective buffers for PO, PPO, β−1,3-glucanase and for gene expression of the corresponding PR proteins as described by Hammerschmidt et al. (1982); Karthikeyan et al. (2005); and Pan et al. (1991) [2,3,5]. The enzyme activity for PO, PPO, β−1,3-glucanase was determined by change of absorbance min-1mg-1 and changes in PR proteins for PR1, PR2, and PR5 were determined using absorbance at 500 nM.
Data source location	• Institution: Tennessee State University, • Department of Agricultural and Environmental Science • Nashville, Tennessee • Country: USA
Data accessibility	Repository name: Harvard Dataverse Data identification number: 10.7910/DVN/YP61IT Direct link to data: https://doi.org/10.7910/DVN/YP61IT
Related research article	E. Rotich, M.T. Mmbaga, J.O. Joshua. Biological control of powdery mildew on Cornus florida using endophytic Bacillus thuringiensis isolate. Can J. of Plant Pathol (2019). https://doi.org/10.1080/07060661.2019.1641555[6].

## Value of the Data


 

• Powdery mildew is a significant constraint in nursery production of flowering dogwood (*Cornus florida*), reducing plant growth and ornamental esthetic value, necessitating routine fungicide applications, and increasing the cost of production [7].
• Although disease resistance is the best option in powdery mildew disease management in nursery production system, lack of good sources of resistance is a problem [7].
• Microorganisms that reside in plant tissue as endophytes are known to protect their host plants through several mechanisms [8], [9], [10], [11].
• Naturally occurring endophytic microorganisms and their products can protect the host plant against powdery mildew infection by inducing host defense enzymes.
• This data is of value to the scientific community by providing information on the role of naturally occurring bacterial endophytes in activating enzymes associated with plant defense as one mechanism of action in microbial-based plant protection.
• This data benefits nursery growers by showing that microbial-based plant defense against powdery mildew may induce plant defense and reduce powdery mildew disease severity in dogwood production.

## 1 Objective

Out of hundreds of microorganisms isolated from flowering dogwood (*Cornus florida*) [8], [9] three bacterial isolates displayed suppressive activity against diverse fungal pathogens and a potential for powdery mildew biological control [10], [11]. The three bacterial isolates B17A (*Stenotrophomonas* sp); B17B (*Serratia marcescens*), and IMC8 (*Bacillus thuringiensis*) suppressed powdery mildew and charcoal rot (*Macrophomina phaseolina*) disease severity in *C. florida* in growth chamber, greenhouse and shadehouse environments [11]. Scanning electron micrographs of dogwood leaves sprayed with bacterial suspensions displayed lysis of powdery mildew spores and hyphae and GC/MS analysis revealed antimicrobial volatile compounds emitted by the bacteria [6]. Analysis of the isolates in the presence of a root rot pathogen, *M. phaseolina*, exposed several volatile compounds that were otherwise not emitted by the bacteria alone indicating that the bacteria triggered volatile compounds against the pathogen [6]. This dataset provides information on plant defense enzyme activities triggered by the bacteria as potential mechanism of action [1].

## 2 Data Description

**Fig. 1:**
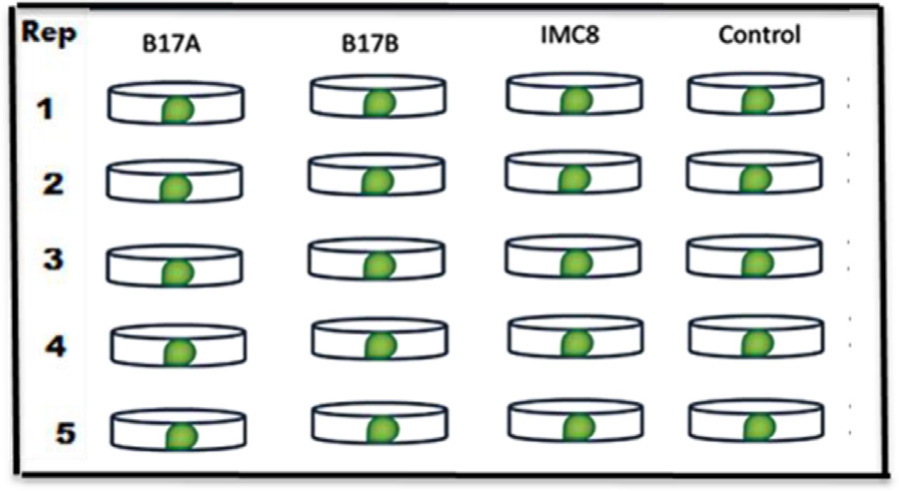
Detached leaf treatments with three bacteria and nontreated control with five replicates and ten leaves per treatment in which B17A (*Stenotrophomonas* sp), B17B (*Serratia marcescens*), and IMC8 (*Bacillus thuringiensis*).

Detached leaves of powdery mildew susceptible flowering dogwood were spray treated with B17A (*Stenotrophomonas* sp); B17B (*Serratia marcescens*), and IMC8 (*Bacillus thuringiensis*) at a concentration of 3 × 10^9^ CFU /ml and negative control sprayed with water. Five replicates per treatment and ten leaves per replicate were used as presented in Fig. 1.

The detached leaves treated with the three bacteria and nontreated control were incubated in moist chambers for 15 h, 26 h, 48 h and 72 h after treatments and analyzed for Peroxidase (PO), polyphenol oxidase (PPO), and β−1,3- glucanase using standard protocols. The gene expression of the corresponding pathogenesis related (PR) proteins and their target genes used Real-time PCR and primers shown in Table 1. The changes in the amount of PR protein were calculated based on absorbance at 500 nM. Data analysis of variance and mean comparisons between treatments was performed using GLM procedure of Statistical Analysis systems (SAS v. 9.4).

**Table 1:** Primers used in the real-time PCR and their target genes.

Primer	Target gene	Primer sequence (5′==>3′)
PR1 F	PR1 (acidic unknown protein)	TTCTTCCCTCGAAAGCTCAA
PR1 R	PR1	AAGGCCCACCAGAGTGTATG
PR2 F	PR2 (β−1,3- glucanase)	AGCTTAGCCTCACCACCAATGT
PR2 R	PR2	CCGATTTGTCCAGCTGTGTG
PR5 F	PR5 (thaumatin like protein)	TGTTCATCACAAGCGGCATT
PR5 R	PR5	GTCCTTGACCGGCGAGAGTTAATGCCGC
At1 F	Internal control (UBQ5)	CACGCTTCATCTCGTCC
At1R	UBQ5	GTAAACGTAAGGTGAGTCCA

**Fig. 2:**
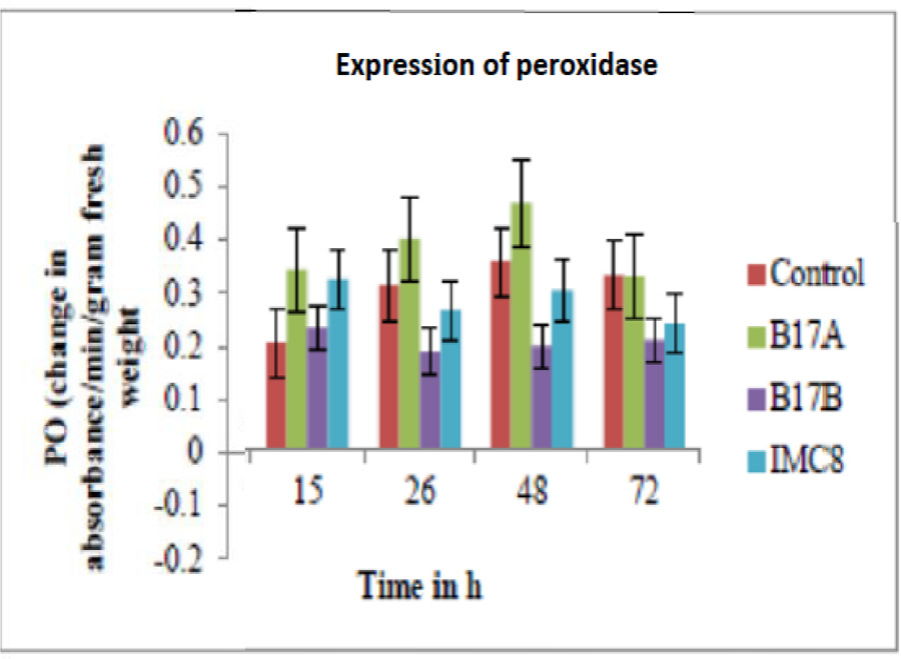
Peroxidase (PO) activity expressed as change in absorbance at 480 nm per min per gram fresh weight of leaves. Control is non-treated, B17A (*Stenotrophomonas* sp.); B17B (*Serratia marcescens*), and IMC8 (*Bacillus thuringiensis*) at 3 × 10^9^ CFU /ml and incubated for 15 h, 26 h, 48 h and 72 h after treatments.

**Results on enzyme activity of Peroxidase (PO):** The highest activity was recorded at 48 h post inoculation, after which there was a decline. The effect of B17A on PO activity was observed as significantly higher than IMC8, B17B and nontreated control (Fig 2).

**Enzyme activity of PPO:** There was an increased PPO activity in leaves treated with

**Fig. 3:**
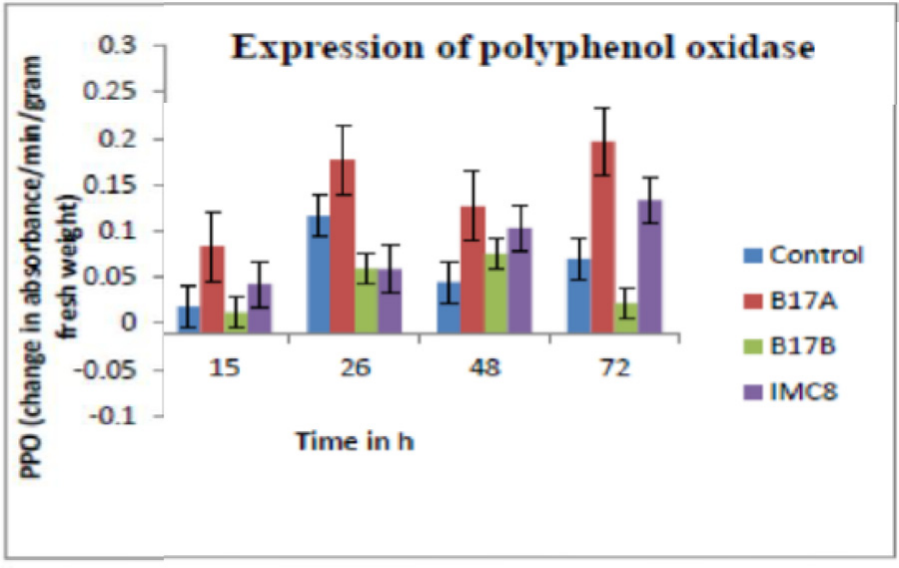
Polyphenol oxidase (PPO) activity expressed as change in absorbance at 480 nm per min per gram fresh weight of leaves. Control is non-treated, B17A (*Stenotrophomonas* sp.); B17B (*Serratia marcescens*), and IMC8 (*Bacillus thuringiensis*) at 3 × 10^9^ CFU /ml and incubated for 15 h, 26 h, 48 h and 72 h after treatments.

B17A as compared to the other two bacterial treatments and water control. The treatment with B17B and IMC8 did not yield a significantly different activity as compared to the water control (Fig 3).

**Fig. 4:**
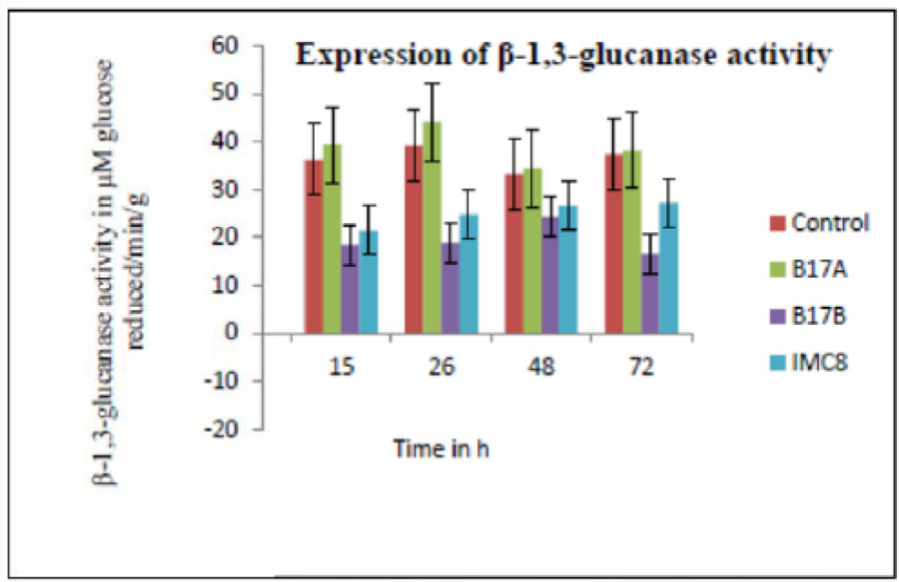
The mean β−1,3-glucanase activity expressed as change in absorbance at 480 nm per min per gram fresh weight of leaves. Control is non-treated, B17A (*Stenotrophomonas* sp.); B17B (*Serratia marcescens*), and IMC8 (*Bacillus thuringiensis*) at 3 × 10^9^ CFU /ml, incubated for 15 h, 26 h, 48 h and 72 h after treatments.

**β−1,3- glucanase activity:** There was no increase in Beta glucanase activity in leaves treated with B17A compared to none treated control and there was no significant difference in the amount of β−1,3-glucanase in IMC8 and B17A treated samples (Fig. 4).

## 3 Pathogenesis Related (PR) Proteins Using Real-Time PCR Assay

The mean Ct (UBQ5) for the internal control gene was 10. The gene expression of the corresponding PR protein for each treatment using the following formula:

$$\begin{array}{ccc} 2-\Delta \Delta \text{Ct} & = & 2[\left(\text{Ct}\text{gene}\text{of}\text{interest}-\text{Ct}\text{internal}\text{control}\right)\;\text{sample}\\ & & -\left(\text{Ct}\text{gene}\text{of}\text{interest}-\text{Ct}\text{internal}\text{control}\right)\text{sample}B] \end{array}$$

**Fig. 5:**
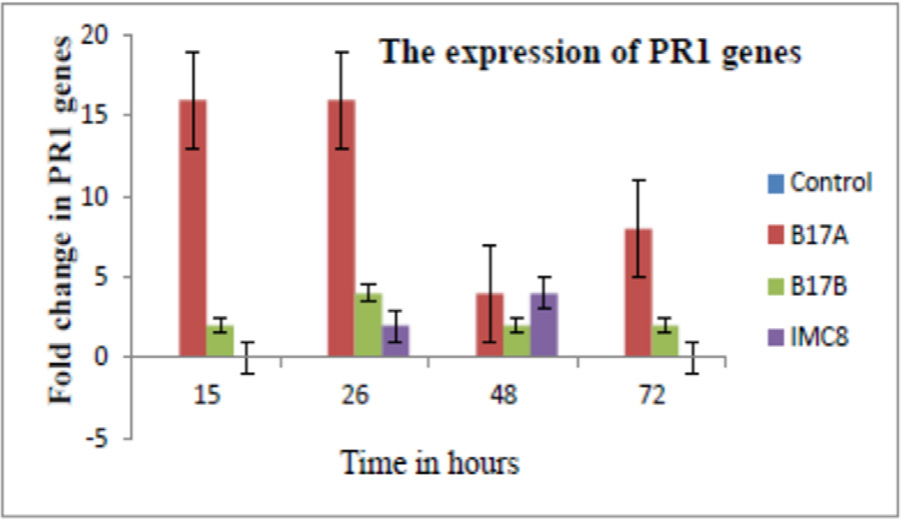
The expression of PR1 genes shown by increases at 15, 26, 48 and 72 h post inoculation with B17A (*Stenotrophomonas* sp); B17B (*Serratia marcescens*), and IMC8 (*Bacillus thuringiensis*) and non-treated (Control).

Showed a16 fold increase in PR1 expression compared to non-treated control at 15 and 26 h post inoculation (pi) in B17A treatment. The increase declined at 48 and 72 h post inoculation as shown in Fig. 5. There was 2 and 4 times increase in PR1 observed in B17B treatments at 15 and 26 h pi respectively as compared to non-treated control. The PR1 expression of IMC 8 was twice the amount at 48 h but was not detected at 72 h pi. There was negligible expression of PR2, and PR5 in all the treatments as compared to control).

## 4 Experimental Design, Materials and Methods

**Detached leaf assay**: This was done in randomized complete block design. The detached leaf assay used leaves from 5 to 6 months old non-treated seedlings and leaves were detached on the day of experiment and placed in 60 mm diameter Petri dishes with 15 mm depth. Prior to experiment 3 × 10^9^ CFU /ml bacterial cultures were prepared in nutrient broth from an overnight growing culture. The experiment used five replicates per treatment and 10 leaves per replicate (Fig 1).

The leaves were incubated in bacterial culture for 15 h, 30 h, 48 h and 72 h. After each incubation period, leaves were harvested, ground in liquid nitrogen, and stored at −70°C until further biochemical assay for enzyme activity and PR proteins.

### 4.1 Experimental Evaluation of Enzyme Activity

Plant extracts were obtained using 1 g of the ground leaf, extracted with 2 ml of 0.1 M sodium phosphate buffer (pH7.0) at 4°C [2] at 15 h, 26 h, 48 h and 72 h post inoculation (pi). The homogenate was centrifuged at 10,000 rpm for 15 min at 4°C, and supernatant used to test for enzyme activity.

**Peroxidase (PO) assay:** The reaction mixture of 1.5 ml 0.05 M pyrogallal, 0.5 ml enzyme extract and 0.5 ml of 1% H2O2 was incubated at room temperature. Change in absorbance was recorded at 420 nm for 3 min at 30 s intervals. The enzyme activity was expressed as change in absorbance min-1mg-1 protein.

**Polyphenol oxidase (PPO) assay:** One gram of the leaf sample was homogenized in 2 ml of 0.1 M sodium phosphate buffer (pH6.5) and centrifuged at 10,000 rpm for 15 min at 4°C. A reaction mixture consisting of 200 µl of enzyme extract, and1.5 ml of 0.1 M sodium phosphate buffer was prepared. A 200 µl of 0.01 M catechol was added to start the reaction and the enzyme activity were measured at 480 nm as change in absorbance min1 mg-1 protein.

**β −1,3-glucanase assay:** One gram of the leaf sample was homogenized with 2 ml of 0.05 M sodium acetate buffer (pH5) and centrifuged for 15 min at 4°C at 16,000 rpm. A reaction mixture was started using the reagents; 62.5 µl, 4% laminarin, and 62.5 µl enzyme extract mixed at 40°C for 10 min. The reaction was be stopped by adding 375 µl of dinitro-salicylic acid, incubating for 5 min in boiling water, then vortexed. The absorbance was measured at 500 nM and the enzyme activity was measured at µg of glucose released min-1 mg-1 protein [3].

### 4.2 Evaluation of PR Proteins: RNA Extraction and RT-PCR Analysis

Total RNA was extracted using RNeasy plant mini kit (Qiagen, Germantown, MD) following the manufacturer's recommendation. The cDNA was transcribed using 1 µg of RNA and subsequently used to amplify respective PR genes using the pathogenesis related primers for PR1, PR2, and PR 5 [4].

Real time (RT)-PCR was performed using ABI Prism (Applied Biosystem, Tokyo, Japan). PCR was carried out in a 20 µl reaction volume containing 1 µl of cDNA, 10 µl of Power SYBR Green Master mix (Applied Biosystems, Warrington, UK), and 0.2 µm of forward and reverse primers (Eurofins, Louisville, KY., Table 1). The mean Ct (UBQ5) for the internal control gene was 10. The gene expression of the corresponding PR protein for each treatment was achieved by using the formula:

$$\begin{array}{ccc} 2-\Delta \Delta \text{Ct} & = & 2[\left(\text{Ct}\text{gene}\text{of}\text{interest}-\text{Ct}\text{internal}\text{control}\right)\text{sample}\\ & & -\left(\text{Ct}\text{gene}\text{of}\text{interest}-\text{Ct}\text{internal}\text{control}\right)\text{sample}B] \end{array}$$

## Ethics Statements

This work meets ethics requirements and does not involve human subjects, animal experiments, or any data collected from social media platforms.

## CRediT authorship contribution statement

**Emily Rotich:** Methodology, Investigation, Writing – original draft. **Margaret T. Mmbaga:** Conceptualization, Visualization, Writing – review & editing.

## Declaration of Competing Interest

The authors declare that they have no known competing financial interests or personal relationships that could have appeared to influence the work reported in this paper.

## Data Availability

Replication Data on bacterial endophytes providing plant defense (Original data) (Dataverse).
